# What Cardiologists Should Know About Amyloidosis

**DOI:** 10.3390/jcm14186668

**Published:** 2025-09-22

**Authors:** Rama Alashqar, Ahmad Alkhatib, Ala W. Abdallah, Mahmoud Odeh, Mustafa Al-Taei, Own Khraisat, Mohammed Al-Hiari, Hazem Taifour, Amer Hammad, Ahmed Sami Abuzaid

**Affiliations:** 1 Washington Hospital Center Program, MedStar Health, Georgetown University, Washington, DC 20010, USA; ramaashqarr@gmail.com; 2 Baltimore Program, Department of Internal Medicine, Medstar Health, Georgetown University, Baltimore, MD 21218, USA; ahmad97khateeb@gmail.com; 3 Kirk Kerkorian School of Medicine, University of Nevada, Las Vegas, NV 89102, USA; ala.abdallah.md@gmail.com; 4 Department of Internal Medicine, Sheikh Tahnoon Bin Mohammed Medical City, Al Ain, United Arab Emirates; mahmoud.odeh99@gmail.com; 5 Department of Cardiovascular Medicine, School of Medicine, Tulane University, New Orleans, LA 70112, USA; mustafataei@outlook.com; 6 Englewood Hospital and Medical Center, Department of Medicine, Englewood, NJ 07631, USA; own.dumam@gmail.com; 7 Jefferson Einstein Philadelphia Hospital, Philadephia, PA 19131, USA; mhmdhiari@outlook.com; 8 Department of Medicine, Unity Hospital, Rochester Regional Health, Rochester, NY 14626, USA; hazemtaifour@gmail.com; 9 Department of Medicine, Banner University Medical Center, Tucson, AZ 85719, USA; amer.hammad.mh@gmail.com; 10 Alaska Heart and Vascular Institute, Anchorage, AK 99508, USA

**Keywords:** amyloidosis, heart failure, hospitalization

## Abstract

**Background:** Cardiac amyloidosis (CA) is an increasingly recognized but historically underdiagnosed cause of restrictive cardiomyopathy and heart failure with preserved ejection fraction (HFpEF). It results from the extracellular deposition of misfolded protein fibrils, most commonly transthyretin (ATTR) or immunoglobulin light chains (AL), leading to progressive myocardial dysfunction and multi-organ involvement. **Objective:** This review provides a comprehensive, cardiology-centered overview of cardiac amyloidosis, with an emphasis on early recognition, diagnostic strategies, subtype differentiation, and the evolving therapies. **Content:** We summarize the epidemiology, pathophysiology, and clinical manifestations of both ATTR and AL subtypes. Key diagnostic tools, including echocardiography, cardiac magnetic resonance imaging, bone scintigraphy, monoclonal protein screening, and endomyocardial biopsy, are reviewed in the context of a stepwise diagnostic approach. Special attention is given to clinical presentation, electrocardiographic and imaging “red flags,” and to differentiating CA from mimickers such as hypertrophic cardiomyopathy, hypertension-induced left ventricular hypertrophy, and aortic stenosis. Staging systems are detailed, highlighting the prognostic role of cardiac biomarkers. Therapeutic strategies are explored, including subtype-specific regimens (e.g., daratumumab-based therapy for AL; tafamidis and gene silencers for ATTR), the judicious use of conventional heart failure medications, and emerging therapies such as CRISPR-based gene editing. **Conclusions:** Timely recognition and accurate diagnosis of cardiac amyloidosis are critical to improving outcomes. As diagnostic tools and disease-modifying therapies evolve rapidly, cardiologists must remain at the forefront of multidisciplinary care. A structured biomarker- and imaging-guided approach can enhance diagnostic yield, inform prognosis, and optimize patient-specific management.

## 1. Introduction

Amyloidosis is a complex disease characterized by extracellular deposition of misfolded protein fibrils, which can result in substantial multi-organ dysfunction. Cardiac involvement is increasingly acknowledged as it leads to heart failure and restrictive cardiomyopathy in affected individuals. Timely diagnosis is essential for optimizing patients’ outcomes, as novel therapeutic agents have shown promise in altering the early course of the disease [1].

This disease presents with a variety of clinical signs and symptoms, although the majority are subtle. Progressive dyspnea, orthostatic hypotension, and peripheral edema are all features that can mimic hypertensive heart disease or hypertrophic cardiomyopathy and other infiltrative cardiomyopathies. The detection of some less reliable findings, such as a low voltage on ECG, raises concern in the appropriate clinical context. Patients can also have ventricular wall thickening that appears out of proportion to the degree and duration of their hypertension. Other red flags described by major cardiology societies include unexplained left ventricular wall thickness ≥14 mm on echocardiogram in patients with heart failure symptoms, discordance between increased wall thickness and low QRS voltage, apical sparing of longitudinal strain, diffuse late gadolinium enhancement on cardiac magnetic resonance imaging (cMRI), and heart failure with preserved ejection fraction (HFpEF) in older adults. Associated conditions such as severe aortic stenosis, bilateral carpal tunnel syndrome, lumbar spinal stenosis, and autonomic or sensory polyneuropathy may further prompt diagnostic evaluation [1,2].

Cardiac amyloidosis is associated with remarkable morbidity and mortality, with the survival of patients depending on the amyloid subtype. AL amyloidosis has a worse prognosis, and if left untreated, has a median survival that is often less than one year due to the rapid involvement of the heart [3]. Fortunately, novel treatments such as TTR stabilizers (like tafamidis and acoramadis) and RNA-silencing therapy have been shown to help increase the survival rate for patients with ATTR amyloidosis. This emphasizes how important early intervention and correct diagnosis really are [4,5]. With increasing recognition and the evolving therapeutic landscape of amyloidosis, cardiologists play an essential role in both early diagnosis and management.

To date, 30–35 proteins have been identified to cause disease in humans, while approximately twice that number are known to form amyloid fibrils in vitro or non-human models. However, the most clinically significant proteins are transthyretin (TTR) and immunoglobulin chains [6]. Transthyretin (TTR), a transport protein synthesized primarily by the liver, plays a central role in transthyretin amyloidosis (ATTR). TTR normally facilitates the transport of thyroxine (T4) and retinol-binding protein but can become destabilized, misfold, and form amyloid fibrils that deposit in various organs, including the walls of the myocardium [4]. This deposition leads to increased ventricular wall thickness, resulting in diastolic dysfunction. On the other hand, misfolded immunoglobulin light chains (ALs) from an abnormal clonal proliferation of plasma cells lead to light-chain amyloidosis.

AL amyloidosis is known to be the more aggressive subtype, as previously mentioned [7]. It predominantly affects males and is associated with multi-organ involvement, including the kidneys, liver, and autonomic nervous system [3]. Cardiac involvement is found in more than 75% of patients with systemic AL amyloidosis at the time of diagnosis and carries a high mortality rate if untreated, often leading to restrictive cardiomyopathy and rapid heart failure progression [8,9].

The hereditary form for amyloidosis is ATTRm, which is caused by mutations in the TTR gene. This leads to unstable TTR tetramers that misfold and ultimately form amyloid fibrils. ATTRm demonstrates variable penetrance and geographic distribution, with certain mutations more common in African-American and Portuguese populations [4]. Cardiac involvement is most pronounced with certain mutations, with infiltrative cardiomyopathy leading to progressive heart failure as the usual clinical condition [10]. On the other hand, ATTRwt, previously known as senile systemic amyloidosis, mainly affects elderly men. It is gradually being recognized as an important factor contributing to HFpEF and aortic stenosis [11]. The disease progresses insidiously, often with a late diagnosis leading to substantial morbidity [12].

Important to note is another subtype of AA amyloidosis that most commonly affects the kidneys, liver, and spleen, with cardiac involvement being uncommon compared to the AL and ATTR subtypes. Studies showed an occurrence of less than 5–10% of AA amyloidosis cases with significant cardiac deposition, which usually is less extensive than other subtypes. The pathogenesis involves chronic elevation of serum amyloid A protein, which can be seen in inflammatory diseases [13,14].

## 2. Diagnostics

A growing body of evidence suggests that all subtypes of amyloidosis remain significantly underdiagnosed, given limited population-level data and persistent diagnostic challenges. Estimates place the AL CA prevalence in the U.S. and Europe between 30,000 and 45,000 cases, with an estimated incidence of 12 cases per million persons annually. Variant ATTRm CA is much less common, with an incidence of about 0.3 cases per million persons per year, with a prevalence of 5.2 cases per million. In contrast, ATTRwt is substantially more prevalent, affecting between 155 to 191 per million [15].

However, delayed recognition often leads to poor outcomes. One review reported that nearly 30% of patients present with advanced, irreversible organ damage and succumb to the disease within months, despite the availability of modern therapies. In particular, diagnostic delays are well documented in light-chain (AL) amyloidosis.

One review reported in fact that diagnostic delays in AL-CA reached up to ≥5 distinct physicians before the correct diagnosis; significantly fewer cardiologists made the correct diagnosis. Most caregivers and patients believed that the correct diagnosis was made most often by hematologists and nephrologists [16].

Transthyretin cardiac amyloidosis (ATTR-CA) also frequently goes unrecognized, with some studies indicating patients undergo a median of 17 healthcare visits before an accurate diagnosis is established [17].

### 2.1. When to Suspect Cardiac Amyloidosis?

When evaluating patients for amyloidosis, both cardiac and non-cardiac cues are essential for heightening the suspicion in the clinical setting. For patients with ATTR cardiomyopathy (ATTR-CM), this role is usually fulfilled by a cardiologist, while a hematologist typically assumes this responsibility for those with AL amyloidosis (AL-CM).

**Figure 1:**
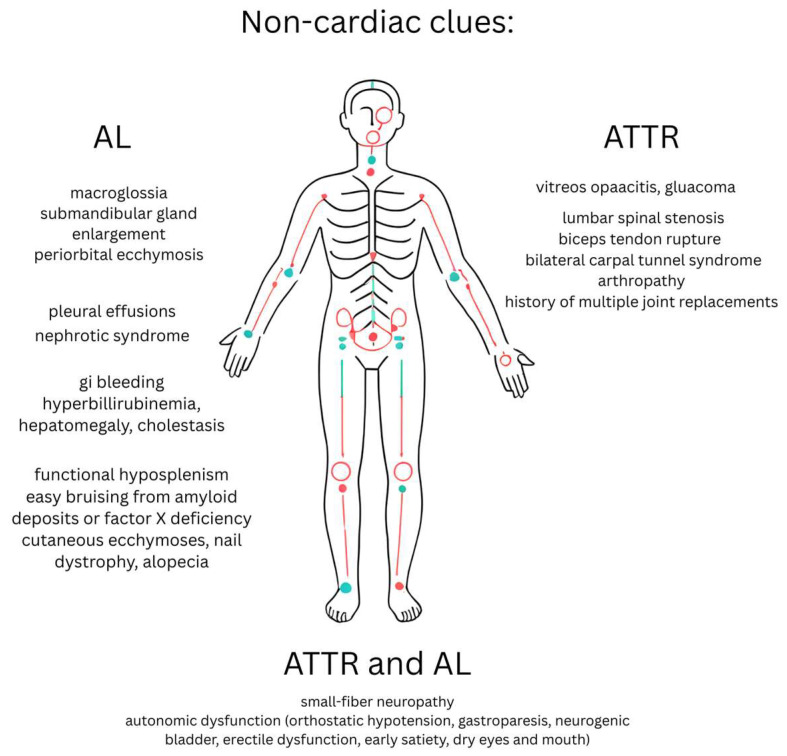
Clinical Manifestations of Amyloidosis.

We will start with non-cardiac clues which are summarized in Figure 1 below.

Interestingly, having bilateral carpal tunnel syndrome in the absence of occupational risk factors was suggested to be an indicator of cardiac amyloidosis as the prevalence of cardiac amyloidosis among these patients was 13.6% based on a study performed by Zegri-Reiriz et al. [13]. Since individuals with amyloidosis typically require follow-up with several specialists, it is crucial to appoint a primary clinician to oversee and coordinate their care. Macroglossia and periorbital bruising on physical examination are almost pathognomonic for AL amyloidosis in the setting of heart failure (HF) [9]. Presence of hepatomegaly and the Kussmaul sign in the absence of added heart sounds and severe valvular dysfunction further raises the suspicion.

As for the cardiac manifestations; they can involve nearly every layer of the heart, from the conduction system to the valves and interatrial septum. The most common early clinical manifestation of the disease is dyspnea on exertion. Coronary microvascular involvement is reflected by persistently elevated troponin levels and reduced peak stress myocardial blood flow (<1.3 mL/g/min), explaining angina in the absence of obstructive coronary artery disease. Myocardial involvement leads to restrictive physiology and signs of both left and right heart failure; in ATTR, asymmetric deposition may mimic hypertrophic cardiomyopathy. NT-proBNP elevations of >5000 (pg/mL) in non-advanced heart failure can also be seen. Valvular involvement includes thickening of the atrioventricular valves and interatrial septum, with paradoxical low-flow, low-gradient aortic stenosis particularly noted in ATTRwt. Electrical abnormalities include but are not limited to low QRS voltage, pseudoinfarct, and atrial fibrillation.

Additional clues include a need to de-escalate or discontinue antihypertensive medications (e.g., beta blockers or renin angiotensin aldosterone system RAAS inhibitors) due to intolerance. This occurs because patients with cardiac amyloidosis often rely on compensatory tachycardia to maintain adequate cardiac output, particularly during exertion, due to stiff, thickened ventricular walls. As a result, blunting this response can lead to inadequate perfusion. These patients may also present with lower-than-expected blood pressure for their age and experience postural hypotension [3,4].

### 2.2. Comparative Pathophysiology of CA, AS, HTN-Induced LVH, and HCM

CA differs in the pathophysiology and the natural history of the disease from other causes of HFpEF, such as hypertension (HTN), hypertrophic cardiomyopathy (HCM), and aortic stenosis (AS). The primary mechanism of the disease is through aggregation and deposition of abnormal amyloid fibrils in the extracellular space surrounding the myocytes, causing an expansion and restriction of normal myocyte function. Clinically, all the aforementioned disorders share similar clinical manifestations and symptoms, which include dyspnea, exertional chest pain, and syncope. CA may also present with symptoms of right HF, including hepatomegaly and ascites, extracardiac manifestations depending on specific organ involvement, and autonomic dysfunction, particularly in ATTR amyloidosis. EKG findings can be used to distinguish between CA and other etiologies. Low QRS voltages are classically seen in CA despite LV thickening, while they are increased in HCM, HTN-induced LVH, and AS [18].

Pseudo-infarct pattern of Q waves also points toward CA, in contrast to deep and narrow Q waves in the lateral leads seen in HCM. Various echocardiographic differences exist between these disorders. The pattern of increased LV wall thickness is diffuse in CA, asymmetric or concentric in HCM, and concentric in both HTN-induced LVH and AS. Diastolic dysfunction is mainly due to restriction in CA, while it is due to impaired relaxation in HCM, HTN-induced LVH, and AS. Hyperdynamic LV systolic function and left ventricular outflow obstruction (LVOTO) are primarily seen in HCM, while the ejection fraction (EF) in CA is usually normal but prone to fall as the disease progresses. GLS classically shows apical sparing in CA, in contrast to uniform global reduction in other disorders. Valvular thickening may be seen in CA and manifests as aortic valve calcification in AS. Pericardial effusion, biatrial enlargement, and granular myocardial appearance are mainly seen in CA as opposed to the different etiologies [19].

AS is not uncommon in amyloidosis patients with an incidence of about 15% and low-flow low-gradient AS of 30%. As the incidence of calcific AS increases with aging and so does the amyloidosis, it can be a challenging diagnosis as the two diseases share many features [20]. Transcatheter aortic valve implantation (TAVI) is the preferred modality over surgical treatment in amyloidosis patients due to the lower perioperative risk when compared to surgical aortic valve replacement (SAVR). Survival rates in both groups are comparable, but TAVI has been utilized more often than SAVR, possibly due to the older age in this population with advanced comorbidities. Overall, both SAVR and TAVI had better outcomes than patients who received medical therapy alone [21].

The prevalence of transthyretin amyloidosis (ATTR) in patients with severe aortic stenosis (AS) is approximately 13–16%. The clinical significance of this prevalence is that there is a need to screen the AS population for ATTR, as it can have a huge impact on clinical outcomes. ATTR is often linked to structural heart diseases such as AS, largely because the two share oxidative stress pathways and fibroblast activation [22]. AS patients with ATTR have an increased risk of heart failure, death, and even aortic valve replacement therapy being futile. In AS patients going through transcatheter aortic valve replacement (TAVR), the presence of ATTR does not have an impact on mortality rate but it will result in higher rates for hospitalization, mainly because of heart failure. Most of the literature on ATTR relates to AS, but limited data exist for other forms of valvular heart disease. Amyloid infiltration can involve any of the heart’s structures, including the valves. ATTR involvement in mitral valves is limited to case studies in the literature. The detection of ATTR in patients with valvular heart disease, particularly those who undergo TAVR, is vital to improve outcomes and optimize management approaches [23].

### 2.3. Diagnostic Work up

Diagnosing cardiac amyloidosis is challenging due to several factors, including its rarity, similarities in presentation with more prevalent conditions that cause myocardial thickening, limited awareness of the appropriate diagnostic algorithm, and the misconception that effective treatments are unavailable.

**Figure 2:**
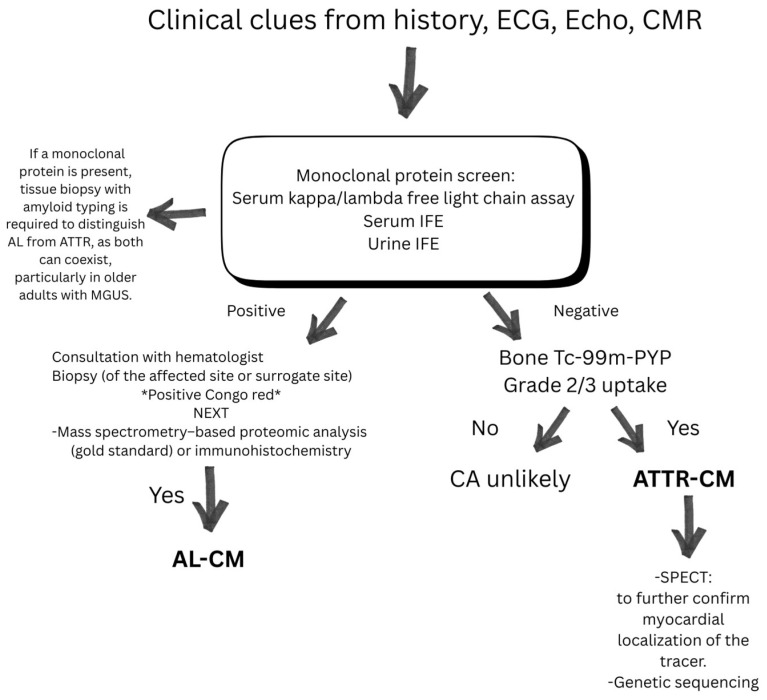
Practical approach for suspected amyloidosis work-up.

Non-invasive testing includes ECG (electrocardiogram), echocardiography, serum free light-chain assay (sFLC), serum immunofixation electrophoresis (SIFE) and urine immunofixation electrophoresis (UIFE), and advanced cardiac imaging such as technetium-99m pyrophosphate scintigraphy (Tc-99m PYP) and cardiac magnetic resonance imaging (CMR). These testing modalities are helpful to diagnose cardiac amyloidosis (Figure 2).

### 2.4. Electrocardiogram

Electrical abnormalities include low-voltage EKGs and disproportionate QRS voltages relative to wall thickness—more commonly seen in AL than ATTR; unusual axis such as extreme right axis deviation are often also present; p-waves are frequently abnormal in morphology and are markedly prolonged; pseudoinfarct (pseudo necrosis) patterns, conduction delays, and atrial fibrillation (especially in ATTR) are all also seen.

As discussed prior, cardiac amyloidosis is an infiltrative process that results in increased myocardial wall thickness, conduction system abnormalities, ECG is an affordable readily available testing modality essential in diagnosis. Classically, ECG with low QRS voltage, especially when combined with paradoxically thickened ventricles, is pathognomonic for amyloidosis. However, studies have shown low-voltage QRS is a late finding in the disease process with a prevalence of less than 40% [24]. Other findings on ECG include a pseudoinfarct pattern, which was found in 83% of a subset of patients diagnosed with cardiac amyloidosis [25]. Conduction abnormalities such as atrial fibrillation can also be present, which were found to occur in 69% of ATTR-CM patients [26]. Summarized below in Table 1 are the ECG findings.

### 2.5. Echocardiography

**Figure 3:**
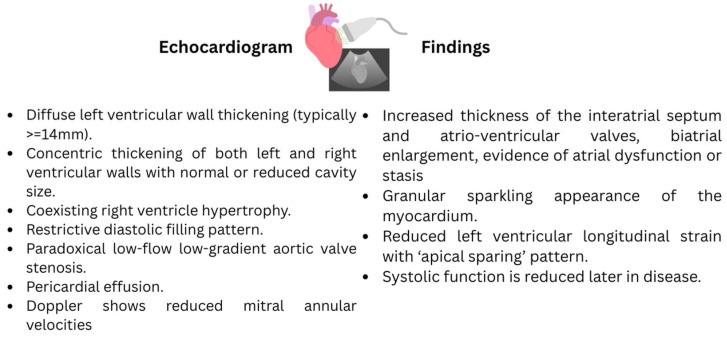
Echocardiographic findings of Cardiac Amyloidosis.

While echocardiography cannot reliably differentiate ATTR from AL amyloidosis, ATTR is often associated with greater wall thickness and lower ejection fraction compared to AL, but both subtypes share the hallmark features described above. The presence of these findings, especially in the absence of long-standing hypertension or aortic stenosis, should prompt further evaluation for cardiac amyloidosis (Figure 3).

The presence of signs and symptoms of cardiac amyloidosis should prompt further testing with echocardiography. Characteristic findings include left ventricular (LV) wall thickening, preserved systolic function, early diastolic dysfunction and elevated filling pressures. The minimal LV wall thickness threshold to screen for cardiac amyloidosis has been lowered to 12 mm by the 2021 European consensus statement [27]. Other possible findings on echocardiography include normal to small LV cavity size, biatrial enlargement and dysfunction, left atrial appendage stasis and/or thrombi, thickening of valves, right ventricular thickening, pericardial and pleural effusions, and a restrictive transmitral doppler filling pattern with tissue doppler imaging tracing, classically showing the “5-5-5” sign (reflecting isovolumetric contraction time, isovolumetric relaxation time, and early rapid filling time) [28]. The “5-5-5” sign arises due to stiff, infiltrated ventricular walls that impair relaxation and filling, leading to a rapid but abruptly halted diastolic filling phase. Diastolic dysfunction is universal, progressing from impaired relaxation to a restrictive filling pattern (E/A > 2, deceleration time < 150 ms) as the disease advances. 

### 2.6. Pulsed-Wave Tissue

Doppler shows reduced mitral annular velocities (e′ and s′ < 8 cm/s), and the E/e′ ratio is elevated [29]. Global longitudinal strain has developed as an emerging tool to predict diagnosis and outcome accurately. Furthermore, apical sparing of the longitudinal strain is highly suggestive of CA [30]. The majority of these features are late findings in the disease process. The classic appearance of starry sky or granular sparkling of myocardial walls are indicative of increased echogenicity of the myocardium, which can also be seen but is insensitive in isolation.

Granular sparkling appearance of the myocardial wall is not sensitive nor specific for CA if present as an isolated finding on echocardiography [31]. Echocardiographic findings in AL are like that of ATTR-CM with a few differences. Increased LV wall thickness is usually less pronounced in AL compared to ATTR amyloidosis and rarely exceeds >18 mm. RV thickening is more frequently observed compared to ATTR-CM. Pericardial effusions are common but are usually small [1].

### 2.7. Cardiac Magnetic Resonance (CMR)

**Figure 4:**
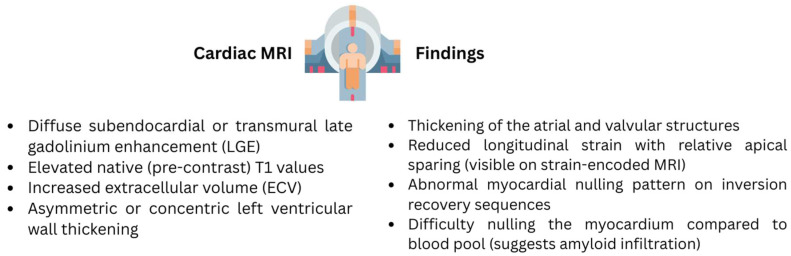
Cardiac MRI findings of Cardiac Amyloidosis.

CMR offers detailed structural visualization and tissue characterization that often supplements the findings from echocardiography, especially in cases when differentiation from other forms of cardiomyopathy is warranted. Key imaging biomarkers or techniques include T1- and T2-weighted imaging, T1 mapping (pre- and post-contrast), late gadolinium enhancement (LGE), and extracellular volume (ECV) imaging. Among these biomarkers, T1 mapping, ECV expansion, and LGE are the most clinically relevant and widely used for diagnosing CA (Figure 4).

LGE imaging in CA shows a typical pattern of diffuse subendocardial or transmural LGE, which is not commonly seen in other cardiomyopathies and is a reliable, non-invasive marker for detecting cardiac involvement. The introduction of phase-sensitive inversion recovery has reduced operator dependency, interobserver variability, with improving consistency [32]. LGE has demonstrated a sensitivity of 86% and a specificity of 92% for detecting CA and appears to be more prevalent in ATTR-CM vs. AL-CM. Pre-contrast (native) T1 mapping measures the T1 signal intensity from both myocytes and extracellular volume as a combined value without gadolinium contrast, which can be particularly useful in patients with renal impairment. Signal is elevated in areas of amyloid infiltration with 92% sensitivity and 91% specificity to detect CA. Additionally, it detects early amyloid deposition in hereditary ATTR mutation carriers before LGE appears [28]. ECV quantifies the extracellular space after gadolinium contrast which is derived from post-contrast T1 mapping. ECV is markedly elevated in both AL and ATTR CA, can correlate with disease severity, and can track treatment response, particularly in AL amyloidosis. It is considered a sensitive early marker in biopsy-proven amyloidosis. However, it lacks specificity in non-biopsy-confirmed cases and cannot distinguish between ATTR and AL subtypes [33,34].

Additionally, LV wall thickness thresholds on steady-state free precession cine CMR can aid in diagnosis—measurements at the mid-cavity level above the upper limit of normal 8 mm (men) and 7 mm (women) on the short axis and 9 mm/7 mm on the long axis are considered suggestive of CA [28,35]. Limitations of CMR include interobserver variability, especially in LGE interpretation; challenges in patients with atrial fibrillation, renal dysfunction, and those unable to hold their breath reliably; and non-MRI-compatible devices—although emerging evidence supports safer use in some device carriers. Despite these hurdles, CMR’s distinct capabilities have proven to be valuable in the evaluation of CA but not sufficient to establish diagnosis as a standalone test nor to differentiate between AL and ATTR.

**Figure 5:**
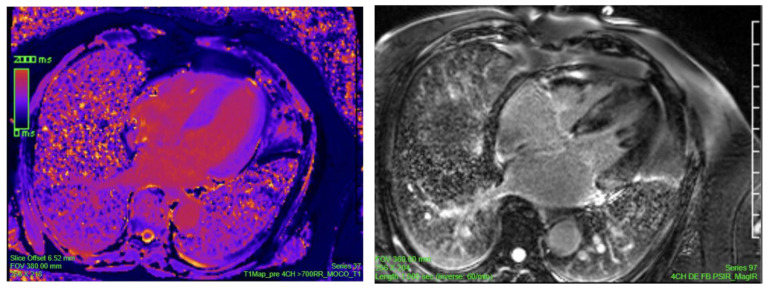
CMR 4 chamber showing patchy appearance of late gadolinium enhancement in the LV septum and papillary muscles with concomitant increased native T1 (1100 ms, mid anterior septum) and ECV (45%), in a patient with biopsy-proven ATTR amyloidosis.

In a patient presenting with ventricular wall thickening and restrictive diastolic filling, the differential diagnosis includes hypertrophic cardiomyopathy (HCM), hypertensive heart disease, cardiac amyloidosis, and Fabry cardiomyopathy. Cardiac MRI with late gadolinium enhancement (LGE) aids in distinguishing among these conditions: amyloidosis typically shows diffuse, global LGE; HCM often demonstrates patchy, mid-wall LGE, particularly in the septum; and Fabry disease characteristically presents with basal inferolateral mid-wall LGE. Although the presence of renal dysfunction may point toward hypertensive cardiomyopathy, Fabry disease or amyloidosis should also be considered, especially when clinical context supports their likelihood. In younger patients with biventricular thickening and restrictive physiology, genetic transthyretin (TTR) amyloidosis, Fabry disease, and hypertrophic cardiomyopathy with a restrictive phenotype should all be included in the differential [36] (Figure 5).

### 2.8. Monoclonal Protein Screening

AL amyloidosis develops due to overgrowth of clonal plasma cells in the bone marrow which results in monoclonal protein presence in serum or urine. Thus, the monoclonal protein screen is an essential tool to screen for plasma cell disorders which can be indicative of AL-CM. It is comprised of three laboratory testing: serum free light-chain (FLC) assay measuring ratio of kappa and lambda light chains, serum immunofixation electrophoresis (SIFE), and urine immunofixation electrophoresis (UIFE), and it is the initial test of choice when high clinical suspicion for cardiac amyloidosis is present based on history, ECG, echocardiography, or CMR findings. The serum FLC assay is positive if abnormal kappa/lambda FLC ratio is present (<0.26 or >1.65) in the setting of normal renal function; patients with renal failure have a suggested normal range from 0.37 to 3.1 [37]. Etiology is due to FLCs being cleared through two primary mechanisms: the kidneys and the reticuloendothelial system. While the reticuloendothelial system removes both kappa and lambda FLCs at similar rates, renal clearance is predominantly more efficient on clearing kappa FLCs [38]. A recent population-based analysis from the iStopMM study proposed new eGFR-stratified reference ranges for serum FLC ratios in patients with chronic kidney disease (CKD), highlighting that current reference intervals may misclassify a substantial proportion of CKD patients due to kidney function-related shifts in FLC clearance. Thus, the authors proposed new eGFR-specific reference intervals for the FLC ratio: 0.46–2.62 for eGFR 45–59, 0.48–3.38 for eGFR 30–44, and 0.54–3.30 for eGFR < 30 mL/min/1.73 m^2^ [39].

SIFE and UIFE are each considered positive if monoclonal protein is detected in either; to note here, serum protein electrophoresis (SPEP) and urine protein electrophoresis (UPEP) are different tests and are insufficient testing modalities for AL amyloidosis as immunofixation is necessary for screening. SPEP was negative in 30% of patients with AL amyloidosis [37]. If there is no monoclonal protein on IFE of the serum and urine and the sFLC assay is normal (normal ratio), then the negative predictive value for excluding AL amyloidosis approaches 99%.

The main point that needs to be raised in this section is that all patients undergoing PYP imaging should also undergo serum and urine immunofixation, as well as serum free light-chain testing. The specificity of PYP scanning decreases significantly in the setting of a known or suspected plasma cell dyscrasia. Without ruling out systemic AL amyloidosis—particularly since monoclonal gammopathies, such as monoclonal gammopathy of undetermined significance (MGUS), are common in older adults due to age and other predisposing factors—clinicians risk misinterpreting PYP results. MGUS can coexist with ATTR or produce imaging findings that mimic ATTR amyloidosis [2,8,39].

In their review, Witteles et al. emphasize that clinicians must be cautious not to overlook abnormal lambda light-chain production simply because the kappa/lambda ratio is within the normal range—especially in patients with reduced eGFR. In such cases, the free light-chain ratio may appear falsely normal due to balanced elevations of both kappa and lambda chains, thereby masking true monoclonal excess. For this reason, physicians must carefully assess absolute light-chain values in the context of the clinical picture, rather than relying solely on the ratio cutoff. This underscores the need for expert interpretation and a multidisciplinary approach when evaluating amyloid diagnostic testing, particularly when non-invasive imaging could be misleading and delay appropriate treatment for AL amyloidosis [40].

### 2.9. Nuclear Imaging

**Figure 6:**
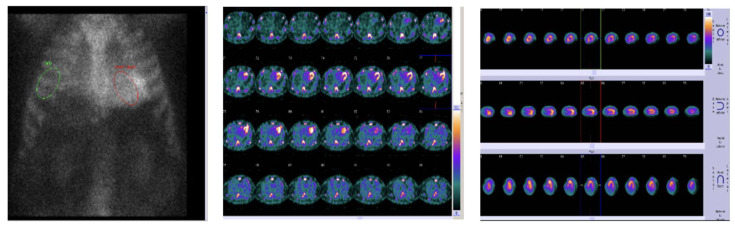
PYP scan for patient with suspected cardiac amyloidosis showing increased heart/lung ration with grade III uptake on SPECT images.

**Figure 7:**
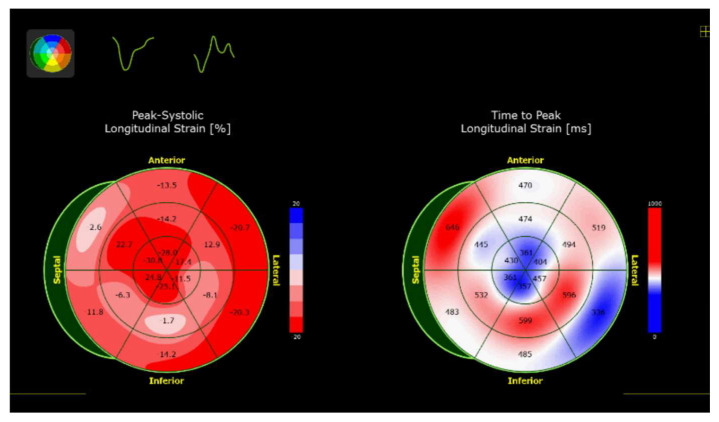
Bull’s eye strain map showing apical sparing pattern in a patient with ATTR amyloid.

The use of bone scintigraphy in CA initially originated in 1980s when incidental myocardial uptake of bone tracers was seen in patients with CA. Technetium pyrophosphate (99mTc-PYP) is the most commonly used tracer in the United States. Based on visual cardiac uptake, scoring is conducted: Grade 0 = no uptake, Grade 1 = mild uptake (<bone), Grade 2 = moderate uptake (equal to bone), Grade 3 = intense uptake (>bone). The presence of Grade 2–3 myocardial tracer uptake demonstrated sensitivity of >99% and specificity of 86% for ATTR-CM and when combined with an absence of monoclonal proteins in serum and urine testing increases the specificity to 100% [41]. Up to 40% of AL-CM patients may show slow uptake, especially Grade 1, leading to potential misclassification. [42] (Figure 6 and Figure 7).

### 2.10. Biopsy in Cardiac Amyloidosis: When to Biopsy and What Is the Yield

Endomyocardial biopsy (EMB) remains the gold standard for diagnosing and characterizing cardiac amyloidosis (CA), particularly when non-invasive testing is inconclusive or when AL amyloidosis is suspected. Although imaging advancements—especially bone scintigraphy using 99mTc-pyrophosphate (PYP)—have enabled non-biopsy diagnosis of transthyretin amyloidosis (ATTR) in specific scenarios, biopsy remains critical in the presence of a monoclonal gammopathy or when PYP uptake is equivocal [28]. While abdominal fat pad biopsies offer a lower-risk approach with approximately 70–80% sensitivity for AL amyloidosis, their utility is limited in ATTR cases. Thus, many specialized centers prefer EMB, which provides high diagnostic sensitivity and specificity when performed by experienced operators. EMB is commonly performed from the right ventricle, though transradial left ventricular access is safe [19]. Congo red staining under polarized light reveals classic apple-green birefringence, and further techniques such as immunohistochemistry, electron microscopy, and mass spectrometry enable definitive amyloid typing [43].

EMB also offers prognostic information when paired with right heart catheterization. Hemodynamic profiles in CA frequently demonstrate elevated right atrial, pulmonary artery, and wedge pressures, consistent with restrictive physiology. Right atrial pressure in particular correlates with transplant-free survival and time to adverse outcomes [44]. Notably, a subset of patients may have both AL and ATTR deposits, highlighting the diagnostic necessity of biopsy even in cases with strong imaging findings.

In summary, EMB remains a cornerstone in the workup of cardiac amyloidosis, especially when distinguishing AL from ATTR or when imaging is inconclusive. Its diagnostic yield and role in guiding personalized therapy make it indispensable in the care of these complex patients [31].

### 2.11. Staging Systems

Using troponin T (hs-cTnT) and N-terminal pro, brain natriuretic peptide (NT-proBNP), a Mayo Clinic research team has developed an effective prognostic staging system for AL amyloidosis. In this system, a Stage I patient is one in whom both hs-cTnT falls below 54 lab levels and NT-proBNP below 332 mL. Stage II is assigned if one biomarker exceeds its cutoff, while Stage III is diagnosed when both markers are raised. Median overall survival has lately become closely linked to these stages: 69 months in I, 29 months at II, and only 6 months of life expectancy remaining for those still at Stage III [45].

A further risk stratification for Stage III can be obtained on the basis of the European modified version of the staging system, those whose NT-proBNP < 8500 ng/L are defined Stage IIIA, while those whose NT-proBNP is above this come into stage IIIB. Patients at Stage III, and particularly IIIB, are at extraordinary risk. They face a particularly poor prognosis [46].

In a series of recent trials, reflecting a nearly universal pattern seen in early studies of amyloidosis, overall mortality was 100%. Notably, one of these trials marked the first attempt at light-chain suppressive therapy, a treatment that has remained the standard of care for multiple myeloma for over 40 years. Patients treated with melphalan and prednisone lived more than twice as long as those who received no chemotherapy—a doubling of survival from 8 months to 18 months [47].

What is remarkable about AL amyloidosis is that, although it originates as a malignancy of the hematologic system, the prognosis is strongly influenced by the degree of cardiac involvement than by any other single factor. The Mayo Clinic developed a scoring system based entirely on biomarkers of myocardial injury: troponin and NT-proBNP. This three-stage system showed median survival times of 2.2, 0.9, and 0.3 years, respectively. A 2012 revision introduced the difference in serum free light chains (≥18 mg/dL) as a third variable, expanding the model into a four-stage system. Median survival improved across all groups, with survival times of 7.8, 3.4, 1.2, and 0.5 years, respectively, reflecting the benefit of modern therapy.

The most recent staging system was introduced by Boston University in 2019. It used BNP rather than NT-proBNP and troponin I as biomarkers. Patients were categorized into four groups: Stage 1 (BNP < 81 pg/mL), Stage 2, Stage 3, and Stage 3b (BNP > 700 pg/mL). At 1- years, median survival for Stage 1 had not been reached; for Stage 2, it was 9.4 years; and for Stage 3, 4.3 years. Because of the relatively recent introduction of these therapies, longer-term outcomes remain uncertain. The free light-chain difference was no longer included in this model, likely reflecting improved control of light-chain production with modern treatment regimens.

Across all staging systems, one central theme emerges: the severity of cardiac involvement is the primary prognostic factor in AL amyloidosis. Prognosis can be accurately stratified by cardiac troponin and natriuretic peptides such as BNP or NT-proBNP. Moreover, the advent of modern therapies has significantly improved survival. However, current staging models may underestimate life expectancy, as most survival data are based on patients who began treatment before newer light-chain suppressive strategies were widely adopted [45,48,49].

Prediction models have emerged as valuable tools for the early diagnosis of transthyretin cardiac amyloidosis (ATTR-CA). The T-Amylo model, incorporating age, sex, carpal tunnel syndrome, interventricular septal thickness, and low QRS voltage, demonstrated strong accuracy in both derivation and validation cohorts (Area Under Curve 0.92 and 0.84) and retained performance across subgroups such as hypertensive cardiomyopathy, severe aortic stenosis, and HFpEF.

Comparative studies suggest the ATTR-CM score offers higher diagnostic accuracy than T-Amylo, while models integrating echocardiographic markers (e.g., wall thickness, longitudinal strain) or multimodal parameters (e.g., relative wall thickness, E/e′, low limb lead voltage) have shown similarly high accuracy for biopsy-proven disease. The 2023 ACC Expert Consensus emphasizes integrating such validated models with clinical, imaging, and laboratory data to enhance early detection and guide workup. After calculating the ATTR-CM score (Table 2), if the score is 6 or higher, the next step is to obtain ATTR-CM scintigraphy. If the scintigraphy result is positive, it is essential to rule out AL amyloidosis and familial amyloidosis [50].

## 3. Treatment

Patients with cardiac amyloidosis have a uniquely compromised myocardium due to extensive amyloid infiltration. This deposition disrupts the heart’s normal architecture, resulting in a stiff, noncompliant ventricle with impaired diastolic filling that reduces stroke volume and eventually progresses to systolic dysfunction in later stages. To compensate, these patients often rely on a higher heart rate to maintain adequate cardiac output. Autonomic dysfunction is also common, further limiting the heart’s ability to adapt to changes in preload and afterload. Together, these pathophysiologic changes create an environment in which the hemodynamic effects of many cardiovascular drugs are exaggerated, and even standard doses can provoke deleterious responses. Thus, expert consensus advises that standard heart failure treatments should be used with caution in patients with cardiac amyloidosis. Additionally, the amyloid burden frequently involves the conduction system, leading to delays, arrhythmias, and even complete blocks [51].

Building on the recognition of autonomic dysfunction, consensus guidelines emphasize treating orthostatic hypotension with a combination of nonpharmacologic and pharmacologic interventions. Supportive measures include compression stockings (knee- or thigh-high), abdominal binders, and increased fluid intake. When pharmacotherapy is required, the ACC recommends midodrine, droxidopa, and pyridostigmine. Salt tablets and fludrocortisone may be considered in select cases, but both are often poorly tolerated due to fluid retention and potential worsening of heart failure. Importantly, deprescribe, or reduce the dose of vasodilators, RAAS inhibitors, and beta blockers in patients with symptomatic hypotension. Overall, management should be individualized and carefully balanced against comorbidities and treatment goals [52].

### 3.1. Diuretics

Diuresis plays a critical role in managing congestion in patients with cardiac amyloidosis, yet its implementation requires a delicate balance. Given the impaired diastolic dysfunction, patients with cardiac amyloidosis are highly dependent on adequate preload to maintain cardiac output and have a narrow euvolemic window. As a result, aggressive diuresis can quickly lead to intravascular volume depletion, precipitating hypotension and further compromising already limited cardiac performance. Moreover, excessive diuresis may reduce renal perfusion, increasing the risk of acute kidney injury in a population that often has concurrent renal dysfunction. Therefore, when employing diuretics—most commonly loop diuretics—in these patients, it is essential to start at low doses and adjust the regimen cautiously [2]. Close monitoring of clinical parameters, renal function, and electrolyte levels is vital to tailor the treatment to each patient’s unique hemodynamic status. This careful approach aims to alleviate symptoms of congestion while preserving sufficient intravascular volume and ensuring that cardiac output is not further diminished by the treatment itself [50,52].

### 3.2. Beta Blockers

Beta blockers, routinely used in heart failure, reduce heart rate and blunt sympathetic stimulation. This decrease in beta-1 receptor activity on the kidneys lowers renin release, which, in turn, limits angiotensin II production, reducing vasoconstriction and fluid retention. It was hypothesized that in amyloid cardiomyopathy, where the heart compensates for low stroke volume by maintaining a higher rate, beta blockers may further reduce cardiac output by slowing the heart rate and impairing the compensatory sympathetic response in response to its vasodilatory effects associated with RAAS inhibition leading to hypotension and decreased tissue perfusion.

However, large observational data showed contrary data regarding its mortality benefit, mostly ATTR. Beta blockers were given to ~half of patients with mixed tolerability; overall, they were not linked to lower all-cause mortality, though low-dose beta blockers were associated with reduced mortality when LVEF ≤ 40%. These signals are vulnerable to selection/survivor bias, so prospective randomized trials are still needed [53,54,55].

### 3.3. Calcium Channel Blockers

Non-dihydropyridine calcium channel blockers such as verapamil and diltiazem are similarly problematic; their negative inotropic effects, compounded by the potential for these agents to bind to amyloid fibrils, can lead to significant hypotension and further compromise myocardial function [31].

### 3.4. Angiotensin-Converting Enzyme/Angiotensin Receptor-Neprilysin Inhibitors

ACE/ARN inhibitors function by blocking the conversion of angiotensin I to angiotensin II, thereby reducing systemic vascular resistance and lowering blood pressure. In conventional heart failure, these effects can lead to improved forward flow and reduced myocardial workload. However, in patients with cardiac amyloidosis, the compromised myocardium relies on the relatively high filling pressures and heart rates to maintain adequate cardiac output. Autonomic dysfunction, a frequent accompaniment of amyloid infiltration, further predisposes these individuals to exaggerated hypotensive responses. In this delicate setting, even modest reductions in afterload can precipitate significant drops in blood pressure, undermining tissue perfusion. While the theoretical benefits of afterload reduction remain appealing, the narrow hemodynamic reserve in cardiac amyloidosis demands that ACE inhibitors be initiated at very low doses and titrated with extreme caution. In some cases, the risk of inducing hypotension and further compromising cardiac output may outweigh the benefits, prompting clinicians to consider alternative therapeutic strategies. This careful, individualized approach is essential to mitigate the potential adverse effects while striving to achieve the beneficial neurohormonal modulation associated with ACE inhibition.

### 3.5. Mineralocorticoid Receptor Antagonists

Recent investigations into the role of spironolactone in cardiac dysfunction have yielded promising insights. A retrospective analysis of the TOPCAT trial, based on patients identified by echocardiographic features (without confirmed amyloidosis), demonstrated that spironolactone was associated with a reduction in the combined endpoint of cardiovascular death, heart failure hospitalization, or aborted cardiac arrest [56]. Additionally, the HOMAGE trial used a machine learning-derived echocardiographic algorithm to identify subgroups at high risk of developing HFpEF who might benefit from spironolactone; in these specific phenotypes—characterized by diastolic changes and structural remodeling—the drug significantly reduced the E/e’ ratio and B-type natriuretic peptide (BNP) levels [57]. Furthermore, a pooled analysis of three randomized trials (HOMAGE, Aldo-DHF, and TOPCAT) evaluated the impact of spironolactone on key echocardiographic parameters in HFpEF patients, demonstrating improvements in the left atrial volume index (LAVi), left ventricular mass index (LVMi), interventricular septum (IVS) thickness, E/e’ ratio, and left ventricular ejection fraction (LVEF) [58]. These findings collectively suggest that spironolactone not only reduces adverse clinical outcomes but also favorably modifies structural and functional cardiac remodeling in these patients.

### 3.6. Digoxin

Digoxin use in cardiac amyloidosis poses unique challenges due to increased sensitivity that may be related to its high affinity for amyloid fibrils, as shown in a 1981 in vitro study with AL and AA fibrils [59]. This binding could result in elevated local drug concentrations, potentially triggering cardiomyocyte receptors even when plasma levels remain low, a mechanism not yet confirmed in vivo for ATTR fibrils. Such unpredictability likely contributes to a higher risk of toxicity and arrhythmias at doses that are generally safe in other forms of heart failure. Consequently, the American Heart Association advises against digoxin use in patients with amyloidosis. Supporting this caution, a retrospective study by Donnelly et al. found that 12% of 69 cardiac amyloidosis patients experienced suspected digoxin-related arrhythmias and toxicity, emphasizing the need for careful patient selection and close monitoring [60]. Similarly, Muchtar et al. reported significant arrhythmic events in 11% of systemic light-chain (AL) amyloidosis patients receiving digoxin, highlighting the importance of using low doses and regularly monitoring drug concentrations, electrolytes, and renal function [61].

### 3.7. Anti-Arrhythmics

Finally, many antiarrhythmic medications have inherent negative inotropic properties or may further disturb conduction in a myocardium already affected by amyloid infiltration. Amiodarone is generally the preferred antiarrhythmic for both rate and rhythm control in cardiac amyloidosis in managing atrial fibrillation, due to its efficacy and tolerability [52]. As mentioned above, beta blockers, digoxin, and calcium channel blockers can provoke deleterious effects. Thus, their use requires a careful balance between controlling arrhythmias and maintaining sufficient contractile function.

In summary, the profound alterations in myocardial structure and function seen in cardiac amyloidosis necessitate a tailored pharmacologic approach. Each drug must be introduced at a low dose and titrated cautiously, with vigilant monitoring to prevent adverse hemodynamic effects and ensure that the fragile balance of cardiac function is maintained.

### 3.8. Specific AL Amyloidosis Treatment

A combination of proteasome inhibitor (Bortezomib), an alkylating agent (Cyclophosphamide), and a steroid (prednisone, or now widely used Dexamethasone) has been used as treatment for AL amyloidosis and showed an excellent hematologic response of about 60% [62]. The ANDROMEDA trial in 2021 introduced Daratumumab, a CD38 monoclonal antibody, as an addition to the previously utilized regimen of CyBorD, showing a significant improvement in the hematologic response of AL patients with a complete response in about 54% of the Daratumumab combination with CyBorD group vs. 26.9% in the CyBorD-only control group [63].

Another form of management is the autologous stem cell therapy (ASCT), which has also been long used for the treatment of MM for three decades. This has shown major improvement in the management of early diagnosed AL cases with a longer overall survival after complete response [64]. An eligibility criterion has been developed to identify which patients qualify for this treatment, and it was shown that only about 20% can undergo this treatment due to the intense induction phase with high doses of chemotherapy in patients with a bone marrow plasmacytosis burden of >10%. Eligible patients are typically younger, with higher functionality levels and a lower extent of organ involvement [65]. Major safety points associated with ASCT are arrhythmia, heart failure, syncope, and end-stage renal disease. However, the high cost of these new therapies poses a significant challenge, and breaking these economic barriers is crucial to ensure broader patient access and maximize the impact of these treatments on survival and quality of life [50].

### 3.9. Specific ATTR Amyloidosis Treatment

ATTR amyloidosis has a more favorable prognosis than AL amyloidosis, with a more diverse profile depending on the identified subtype (wild vs. hereditary), as highlighted in the prognosis section.

Among the other treatments proposed and according to the current guidelines, tafamidis is the only treatment approved for the treatment of ATTR amyloidosis. Tafamidis is a TTR protein stabilizer that prevents the degradation into small monomers and thus prevents amyloid fibrils and slows down the deposition into the tissues [4]. It has shown decreased mortality and cardiovascular hospitalization, as well as improved 6 min walk test and quality of life.

**Figure 8:**
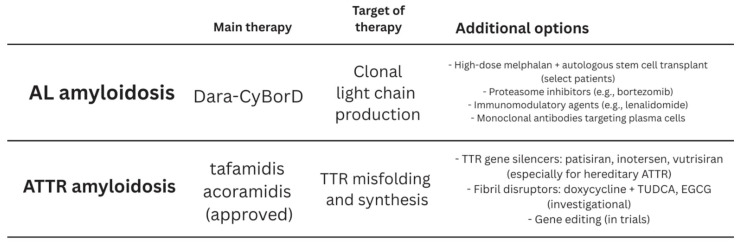
Therapeutic treatments for Cardiac Amyloidosis.

Acromadis, another TTR stabilizer, has shown promising results in the ATTRibute-CM trial, showing efficacy and safety at least comparable to tafamidis, and it is distinguished from emerging therapies by its mechanism, oral administration, and robust clinical trial data supporting its use in both wild-type and variant ATTR-CM [5]. Other treatment options include silencers, small interfering RNA agents, CRISPR-Cas9, and liver transplant [66]. Diflunisal is an alternative TTR stabilizer that can be used off-label for some patients. Nevertheless, it is necessary to keep an eye out for its renal and gastrointestinal side effects. New therapies include TTR gene silencers, such as inotersen or patisiran, in which hepatic TTR synthesis is disrupted through either mRNA breakdown or inhibition. Both of these agents are approved for hereditary ATTR with polyneuropathy and are also under investigation regarding cardiac involvement. Other experimental treatments include doxycycline-terurosodeoxycholic acid (TUDCA or ursodeoxycholic acid). These may interrupt amyloid fibril creation, though whether or not these drugs have any effects at all on clinical outcomes is unknown (Figure 8).

### 3.10. Use of ICDs in CA Patients

ICD therapy is strongly indicated for secondary prevention in patients with sustained ventricular tachycardia (VT) and sudden cardiac death (SCD), which is a class I recommendation. While patients with cardiac amyloidosis have an increased risk for SCD and VT, use of ICDs in this population remains controversial [67]. A retrospective study comparing the use of ICD in CA to non-ischemic cardiomyopathy (NICM) patients showed increased mortality in CA at about 26.9% vs. 11.3% [68]. A meta-analysis showed that some patients with amyloidosis may receive appropriate shock. Still, no clear survival benefit was shown from that study [69,70]. Due to the above conflicting results and lack of randomized trials, no recommendations for generalized use of ICDs exist in these patients, and individual assessments should be carried out [71,72].

### 3.11. LVADs and Heart Transplants in CA

Recent studies have demonstrated the evolving role of heart transplantation in cardiac amyloidosis (CA). Outcomes have improved over the past decade, with 1-year survival approaching that of non-amyloid cardiomyopathies, especially in ATTR patients and carefully selected AL amyloidosis patients. Long-term survival is also feasible, especially when systemic disease is controlled with modern therapies. Combined organ transplantation may be considered for those with significant extracardiac burden; for example, patients with neuropathy due to variant TTR may receive heart–liver transplantation to halt mutant TTR production and prevent the progression of systemic disease.

The use of left ventricular assist devices (LVADs) in CA remains limited. Small LV cavity size and restrictive physiology carries significant challenges, and most published series report inferior outcomes with LVADs alone compared with other etiologies of heart failure. However, mechanical circulatory support can be considered in selected patients as a bridge to transplant.

### 3.12. Prognosis

Cardiac involvement is a key determinant of the prognosis in amyloid patients; however, it is worth mentioning that the mortality rate associated with cardiac amyloidosis has declined in recent years. A study published from Denmark demonstrated that the 2-year mortality rate for cardiac amyloidosis patients decreased from 82.6% in the period 1998–2002 down to 50.2% in 2013–2017 [6].

The median survival for patients with untreated ATTRwt cardiomyopathy ranges from 3.6 to 4.8 years, while it is approximately 2.6 years for ATTRv cardiomyopathy. In contrast, untreated AL cardiac amyloidosis has a median survival from 0.3 to 2.2 years^.^ To put it simply, the later the diagnosis is, the worse the prognosis is. One should have a high clinical suspicion to identify amyloidosis, as its systemic nature affects multiple organs, leading to widespread and often vague symptoms. A heightened clinical awareness of subtle signs and nuances of this disease can facilitate early diagnosis and potentially shift the outcomes curve in a more positive direction [73].

For patients with advanced heart failure and poor overall prognosis and outcomes, advanced therapies should be offered. Heart transplantation is a feasible option when all the therapies have been exhausted, which can prolong survival even in patients with AL cardiac amyloidosis for more than 10 years in select patients [74]. Unfortunately, despite the increase in the use of heart transplantation nowadays and the number of organ donors, the wait can be long, with an ever-rising waitlist mortality [50]. Mechanical circulatory support is an option as a bridge to transplant, keeping in mind that it comes at the cost of a higher mortality rate attributed to the many complications such devices harbor [75]. These treatments, however, are not suitable for everyone; severe multiorgan involvement of amyloidosis, frailty, and advanced age are looked at as negative factors. As a result, transplant centers tend to shy away from offering them advanced therapies due to the impact on post-transplant morbidity and mortality [76]. This fact underscores the importance of early diagnosis and treatment in patients with cardiac amyloidosis. Collaboration with palliative care specialists can be useful to enhance symptom management, guide patients when faced with complex treatment decisions, and provide emotional and psychosocial care [50,72].

### 3.13. Future Research

Promising therapies now under study include Eplontersen, a GalNAc-conjugated antisense oligonucleotide that is being evaluated in the CARDIO-TTRansform trial for its effects on cardiovascular outcomes and mortality, with results anticipated by 2025 at the earliest. NTLA-2001, a pioneering in vivo CRISPR-Cas9 gene-editing therapy for transthyretin (TTR) amyloidosis, aims to achieve permanent suppression of TTR production after a single administration, being assessed in the MAGNITUDE trial. Furthermore, NI006 (ALXN2220) is an antibody designed to target misfolded TTR deposits; following encouraging Phase I results that demonstrated reductions in cardiac amyloid burden and associated biomarkers, it is currently being assessed in the DepleTTR-CM Phase III study. Additionally, NNC6019 (formerly PRX004) is a monoclonal antibody that promotes the immune clearance of amyloid and is now being evaluated in a Phase II trial to determine the optimal dosing regimen and its impact on functional status, cardiac biomarkers, and structural changes. Collectively, these trials signify a significant shift toward disease-modifying and potentially curative therapies for ATTR-CM. These gaps in knowledge, as the ESC Working Group’s position statement on cardiac amyloidosis emphasizes, are vital questions that demand answers by future research and joint consensus building. There is also a lack of dedicated data for how the widely used guideline-directed medical therapy (GDMT) for heart failure with preserved ejection fraction (HFpEF) affects people with this condition—impossible even to begin discussion without more targeted research. And if there were not enough problems already, the escalating number of therapies presents not just technical dilemmas to doctors, but also an urgent social-economic decision: how affordable will they be? How easily reachable [77]? 

Yet, the promise of these therapies underscores an equally critical need for timely and accurate diagnosis: recent advances in AI-based echocardiographic screening have shown excellent ability to differentiate cardiac amyloidosis from phenotypic mimics using only apical four-chamber views, outperforming conventional diagnostic scores. Integrating such tools into practice could ensure that patients are identified earlier and can access these transformative therapies, though questions remain regarding cost, accessibility, and the role of conventional GDMT in this unique population.

## 4. Conclusions

Cardiac amyloidosis has evolved from a relatively under-recognized condition to a dynamic area of study marked by rapid advances in imaging technology, detection markers, and treatments that may indeed change the course the disease takes. The key to successfully treating any disease, including this one, is early diagnosis. Therefore, it is essential to recognize the signs that may point to an existence of this particular disease.

On the other hand, newly developed approaches are being developed for both AL and ATTR subtypes of cardiac amyloidosis, ranging from nuanced stabilizers and sRNA molecules to attractive gene-editing techniques. Experimental therapies may offer hope for these challenging conditions, but they also give rise to formidable questions awaiting answers regarding access and cost, as well as which patients should receive which type of treatment. In the continuing diagnosis or treatment of cardiac amyloidosis, there are still a lot of issues that need to be addressed. The treatment landscape is evolving, and it is crucial to know more about the interaction between silencers and stabilizers as well as any supplementary effects they might have on both heart and kidney protection.

In the years ahead, a multifaceted, patient-oriented approach that begins with early diagnosis and goes on to give comprehensive treatment will be fully behind the improvement of life expectancy for both longevity and quality in those suffering from cardiac amyloidosis.

## Figures and Tables

**Table 1:** Common ECG findings in cardiac amyloidosis.

ECG Finding	Prevalence	Reference
Low QRS voltage	<40% of biopsy-proven cardiac amyloidosis [21].	Cyrille et al. [24]
Pseudoinfarct	83% of a subset of patients diagnosed with cardiac amyloidosis [22].	Roberts et al. [25]
Atrial fibrillation	69% of ATTR-CM patients in a retrospective study [23].	Donnellan et al. [26]

**Table 2:** ATTR-CM score.

Clinical Variable	Value	Points
Age (years)	60–69 = 2; 70–79 = 3; ≥80 = 4	2–4
Sex	Male = 2	2
Ejection Fraction	<60% = 1	1
Posterior Wall Thickness	≥12 mm = 1	1
Relative Wall Thickness	>0.57 = 2	2
Hypertension History	Present = −1	−1

## Data Availability

No new data were created or analyzed in this study.
